# Idiopathic epilepsy in dogs is associated with dysbiotic faecal microbiota

**DOI:** 10.1186/s42523-025-00397-w

**Published:** 2025-03-27

**Authors:** Marco Silvestrino, Mattia Pirolo, Angelica Bianco, Stefano Castellana, Laura Del Sambro, Viviana Domenica Tarallo, Luca Guardabassi, Andrea Zatelli, Floriana Gernone

**Affiliations:** 1 Department of Veterinary Medicine, University of Bari, Str. Prov. Per Casamassima km3, 70010 Valenzano, Italy; 2 Department of Veterinary and Animal Sciences, University of Copenhagen, Frederiksberg C, Denmark; 3 Istituto Zooprofilattico Sperimentale della Puglia e della Basilicata, 71121 Foggia, Italy

**Keywords:** Gut microbiome, 16S rRNA, Canine idiopathic epilepsy, SCFA, Epileptogenesis, Dysbiosis

## Abstract

**Background:**

The gut microbiota plays a crucial role in modulating various physiological and pathological processes through its metabolites, including short-chain fatty acids (SCFA), which impact immune system development, gastrointestinal health, and brain functions via the gut-brain axis. Dysbiosis, an imbalance in gut microbiota composition, has been linked to neuroinflammatory and neurodegenerative conditions, including epilepsy. In dogs, idiopathic epilepsy has been hypothesized to be influenced by gut microbiota composition, although studies on this association are limited and show inconsistent results. Here, we compared the faecal microbiota of idiopathic epileptic drug-naïve dogs and healthy controls. To this aim, we recruited 19 idiopathic epileptic dogs and 17 healthy controls which met stringent inclusion criteria and characterized their faecal microbiome by 16 S rRNA sequencing.

**Results:**

No significant differences were observed between the two groups regarding age, breed, body condition score, diet, or reproductive status, though males were significantly overrepresented in the idiopathic epileptic group. Epileptic dogs showed a marked reduction in bacterial richness and a trend towards lower evenness (α-diversity) compared to healthy controls, while no differences in community composition (β-diversity) were observed between the two groups. Moreover, a decrease in SCFA-producing bacteria, namely *Faecalibacterium*, *Prevotella*, and *Blautia*, was observed alongside an increase in *Escherichia coli*, *Clostridium perfringens*, and *Bacteroides* in epileptic dogs.

**Conclusions:**

Idiopathic epileptic dogs exhibit dysbiosis, with reduced bacterial diversity, loss of beneficial genera, and overgrowth of opportunistic pathogens. These alterations in microbiota diversity and composition may contribute to epilepsy via the gut-brain axis, highlighting the need for further research to explore dietary or probiotic interventions targeting gut microbiota modulation as adjunctive therapies for managing epilepsy in dogs.

**Supplementary Information:**

The online version contains supplementary material available at 10.1186/s42523-025-00397-w.

## Background

The gut microbiota (GM) modulates physiological and pathological processes through direct and indirect interactions with the host, including the producing short chain fat acids (SCFAs) and neurotransmitters, metabolizing dietary derived tryptophan and primary bile acids [1, 2]. Moreover, the GM provides an important support in digestion and energy yield from the diet [3], guarantees the nutritional support for enterocytes [4], acts directly defending the gastrointestinal (GI) barrier against pathogens [5] and stimulate the development of the immune system [6].

SCFAs are the major investigated GM metabolites produced by intestinal bacteria [1, 2, 7], due to their modulatory activity on the host’s immune function, energy storage, integrity of anatomical barriers, and physiological metabolism and functions in the GI tract and distal organs, including the brain [7, 8]. The interaction between the GI and the brain, named gut-brain axis, is modulated by the GM-produced SCFAs, which can cross the blood-brain barrier (BBB) via monocarboxylate transporters located on endothelial cells, contributing to the maintenance of BBB integrity, reducing the cerebral and cerebrospinal fluid concentrations of pro-inflammatory cytokines, supporting the brain metabolism with SCFAs-derived ketogenic bodies, preventing the activation of microglial cells and astrocytes to neuroinflammation, and modulating the levels of inhibitory neurotransmitters [8, 9]. Consequently, both dysfunctional or pathological condition which may alter the GM composition and/or functionality (i.e., dysbiosis), could threaten the host’s brain homeostasis, evolving into neuroinflammatory and facilitating neurodegenerative disorders and epilepsy [8, 9].

Epileptic seizures are defined as the sudden and transient manifestation of an abnormal, synchronous, and self-limited electric activity of cortical neurons, associated with dysfunction of inhibiting neurotransmitters and/or a massive release of excitatory ones [10, 11]. In dogs, epileptic seizures are very common with an estimated prevalence of 0.75–0.82% of the canine population [12, 13]. The International Veterinary Epilepsy Task Force (IVETF) divided causes of seizures into reactive (i.e., consequent to toxic or metabolic disorders), structural (i.e., provoked by intracranial/cerebral pathology) and idiopathic epilepsy, which are defined as the chronic enduring brain predisposition to generate seizures without apparent underlying cause [10]. With a prevalence of 53.8%, idiopathic epilepsy is the most common recorded cause of epilepsy in dogs [14]. It has been hypothesized that the GM and its metabolites may influence the pathogenesis of epilepsy via the gut-brain axis [9, 15, 16], and specific dietary interventions are thought to contribute to the management of idiopathic epilepsy in dogs in addition to standard antiepileptic medical treatments, by modulating GM composition and/or its metabolism [17–19].

The existing correlation between GM metabolism and epileptogenesis is largely based on human studies. To date, only two clinical trials explored the faecal microbiota composition in epileptic drug-naïve dogs [20, 21], and their results are inconsistent due to different research questions (i.e., focusing only on a specific bacterial genus) [20] or to methodological bias (sheltered control dogs of the same breed and fed with the same diet) [21]. The objective of the study was to undertake a comparative analysis of the whole faecal microbiota of untreated idiopathic epileptic and healthy client-owned dogs of different breeds fed a heterogeneous diet, in search of significant differences in the relative abundance of bacterial genera involved in the production of metabolites useful for maintaining brain health.

## Materials and methods

### Animals

Idiopathic epileptic and healthy client-owned dogs of any sex and breed, between 6 months and 6 years old (or have firstly experienced seizures between 6 months and 6 years) presented from June 2021 to May 2023 to the Veterinary Teaching Hospital of the Department of Veterinary Medicine, University of Bari, Italy, were recruited for this study. All owners of enrolled subjects signed an informed consent form to allow the dogs to be included in the study and for the necessary diagnostic procedures to be carried out. Patient information including signalment, clinical history, diet, recent antimicrobial, de-worming and probiotic treatments, diet supplementation, a family history for epileptic dogs were recorded for each dog. A complete physical examination, including body condition score (BCS; based on a 1 to 9 scale with 4–5 being optimal) [22], was performed by a Board-certified neurologist (FG) or PhD student (MS). Dogs were excluded from the study if (a) affected or suspected to be affected by endocrinopathies or dermatopathies or other intestinal or systemic pathological conditions; (b) treated with antimicrobial, de-worming drugs and probiotics in the previous three months; (c) presenting GI signs (i.e. vomiting and/or diarrhoea) in the previous three months; (d) underwent abdominal surgery within the previous 3 months; (e) pregnant and/or in lactation. Moreover, dogs were considered healthy, and eligible for enrolment in the study as a control group, based on unremarkable physical examination and no significant abnormalities on blood, urinary and coprological test.

Epileptic dog owners were asked to share recordings of epileptic episodes as confirmation of epileptic seizures. Diagnosis of idiopathic epilepsy was based on Tier I and partial Tier II confidence level (i.e. urinary bile acids:creatinine ratio) as proposed by IVETF consensus [23].

Complete blood cell count (CBC), serum biochemistry (including alanine aminotransferase, aspartate aminotransferase, alkaline phosphatase, creatinine kinase, total serum protein, with albumin and globulin fractions, urea, creatinine, cholesterol and triglycerides, lipase, calcium, sodium, potassium, chloride, phosphate, total bilirubin, and C-reactive protein), serum protein electrophoresis, and urinalysis (including sediment, pH, specific gravity, glucose, total bilirubin, ketones, urinary bile acids: creatinine ratio, and urinary protein:creatinine ratio) were performed to rule out metabolic or systemic diseases responsible for reactive seizures in the epileptic groups and to confirm the healthy status in the group of control dogs. Coprological examinations were also performed in all enrolled dogs to rule out enteric parasitic infections.

### Sample collection and storage

Blood samples were collected from jugular or cephalic veins in a 1 ml BD Vacutainer K3-EDTA tube (BD Belliver Industrial Estate, UK) to perform CBC, and in a 3 ml BD Vacutainer clot activator serum tube (BD Belliver Industrial Estate, UK) to perform serum biochemistry and serum protein electrophoresis. A minimum of 5 ml of urine samples were collected in a BD Vacutainer urinalysis preservative tube (BD Belliver Industrial Estate, USA) on the date of presentation by spontaneous voiding. All samples were immediately refrigerated at 4 °C and CBC (Siemens, ADVIA 2120, Erlangen, Germany), serum biochemical analysis (Beckman Coulter, Clinical Chemistry Analyzer AU680, Indianapolis, USA), serum protein electrophoresis (SEBIA Italia S.r.l., Capillarys 2 Flex Piercing, Italy), and urinalysis were performed within 24 h from sampling. Urine specific gravity was measured using a refractometer (Leica Vet 360, Misco Products Division, Cleveland, USA). Urine dipstick examination (including pH, glucose, ketone bodies and bilirubin) was interpreted as recommended by the manufacturer (Combur 9 Test, Roche, Switzerland). To determine the urine proteine:cratinine ratio, the protein concentration (mg/dL) was assessed using pyrogallol red-molybdate assay, while the serum creatinine (mg/dL) was measured through the Jaffé method in undiluted urine. Urine sediment was obtained via centrifugation and microscopically assessed.

Faecal samples for microbiome analysis were collected at the time of enrolment and immediately stored at − 80 °C. A second sample was also collected for coprological examination by faecal smear, flotation and Baermann test according to the ESCCAP guidelines [24].

### Faecal microbiota profiling

Total DNA from faecal samples was extracted using the QIAamp UCP Pathogen MiniKit (QIAGEN, Copenhagen, Denmark), with the addition of a bead-beating step using the Pathogen Lysis tube S (QIAGEN), and the inclusion of two blank extraction controls. Quantification of DNA was performed using Qubit 3.0. Fluorometer (ThermoFisher Scientific, Cleveland, OH, USA). The V3-V4 region of the 16 S rRNA gene was amplified using the 16 S Metagenomic Sequencing Library Preparation kit (Illumina, San Diego, CA, United States), according to manufacturer instruction. Sequencing was performed on an Illumina MiSeq platform (2 × 300 bp paired-end reads) using the MiSeq Reagent Kit v3 (600 cycles; Illumina), according to manufacturer’s instructions. Sequencing data have been submitted to the NCBI Sequence Read Archive (SRA) under BioProject PRJNA1159160.

16 S rRNA sequencing data were processed in R v4.2.1 using the DADA2 v1.14.1 pipeline [25], after removal of adapter sequences using cutadapt v3.4. The resulting amplicon sequence variant (ASV) were taxonomically assigned using the Silva database v.138.1 [26] and only sequences assigned to Bacteria were retained. The final phyloseq object was constructed using phyloseq v1.30.0 [27], reads were transformed using the cumulative sum scaling method.

### Statistical analysis

Data analysis was performed in R v4.3.3. Categorical (i.e., sex, diet and reproductive status) and numerical (i.e., age and BCS) variables were compared with Chi-squared test and t-test, respectively. For 16 S rRNA data analysis, alpha-diversity (Chao1 and Shannon indexes) and beta-diversity (Bray–Curtis dissimilarity metric) were calculated using R package vegan v.2.6.4. Comparison of alpha-diversity indexes was performed using the Wilcoxon Rank Sum test and p-values were corrected for multiple comparisons using Holm’s correction. Beta-diversity was visualized using a Principal Coordinates Analysis (PCoA) plot, and differences in beta-diversity were estimated by permutational multivariate analysis of variance (PERMANOVA) using the Adonis function and 999 permutations. Differential abundance analysis between groups was performed using DESeq2 and contrasts were corrected for multiple comparisons using the Benjamini-Hochberg’s correction. Only ASVs with adjusted p-values < 0.05 and estimated fold change > 2 were considered significantly differentially abundant. P-values below 0.10 were regarded as a tendency and p-values below 0.05 were regarded as statistically significant.

## Results

### Study population

Based on physical and neurological examinations 26 suspected IE dogs and 24 supposed healthy control (HC) dogs were initially considered eligible for enrolment. Seven of the suspected IE dogs were excluded based on our predefined exclusion criteria, including elevated urinary bile acids: creatinine ratio (*n* = 2); elevated CRP and leucocytosis (*n* = 1), hypoalbuminemia and hyperglobulinemia with increased alfa-2 serum protein fraction (*n* = 1), suspected protein-losing enteropathy (*n* = 1), only a single episode of seizure presented along the study period (*n* = 1) or suspected paroxysmal dyskinesia (*n* = 1). Seven dogs in the HC group were excluded from the study, as they presented thrombocytopenia and elevated C-reactive protein and urine pH (*n* = 1), thrombocytopenia, hyperglobulinemia and azotemia (*n* = 1), thrombocytopenia and elevated urinary bile acids: creatinine ratio (*n* = 1), lymphocytosis (*n* = 1), an active urine sediment (*n* = 1), thrombocytopenia, elevated C-reactive protein and active urine sediment (*n* = 1), or roundworm positivity (*n* = 1).

**Fig. 1:**
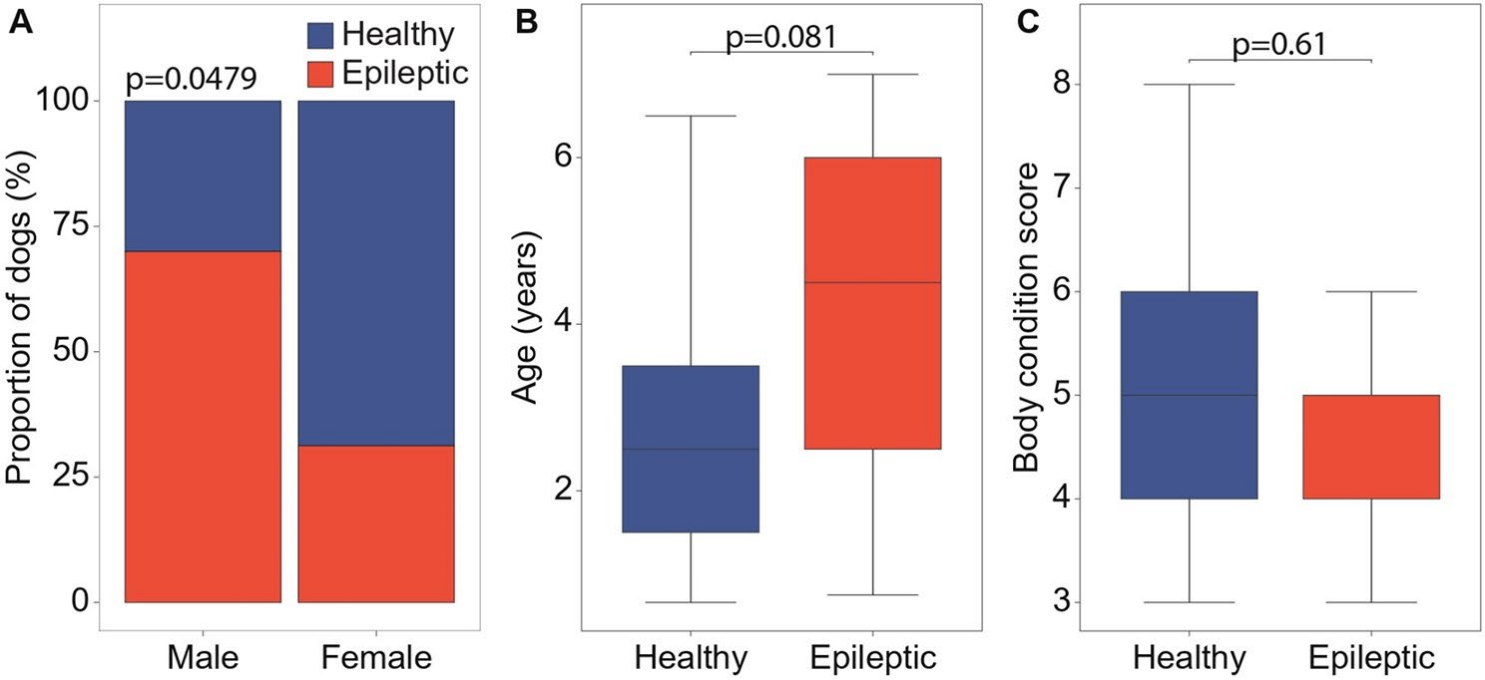
Comparison of sex (**A**), age (**B**) and body conditions score (**C**) between healthy and epileptic dog

The final study population consisted of 19 dogs in the IE group and 17 dogs in the HC group. Breed, sex, reproductive status, age, BCS, and dietary regimens for each dog are presented in Supplementary Table S1. The two groups showed a heterogeneous distribution of breed and no association with diet or reproductive status was observed (*p* > 0.05). Males were significantly overrepresented in the IE group than in the HC group (*p* = 0.0479) (Fig. 1A), while no significant differences in age (mean 4.1 years for IE dogs vs. 2.8 years for the healthy controls, *p* = 0.081; Fig. 1B) and BCS (mean 5.0 in IE dogs vs. 5.2 in healthy controls, *p* = 0.61; Fig. 1C) were observed between the two groups.

### Faecal microbiota composition and diversity

**Fig. 2:**
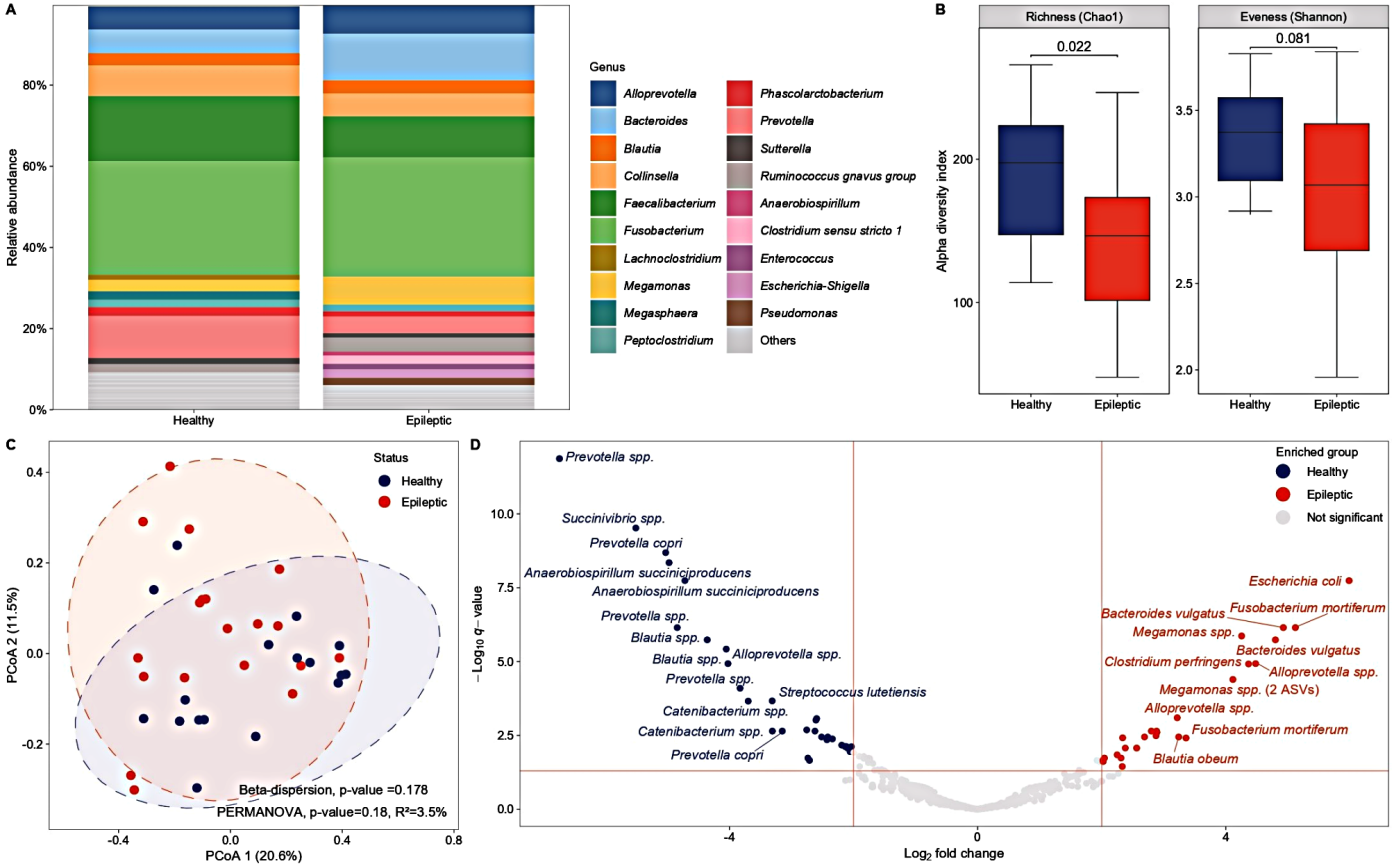
Faecal microbiota comparison of healthy (*n* = 17) and epileptic (*n* = 19) dogs. **A**) Relative microbial abundance (genus level). Only taxa with a relative abundance ≥ 2% are displayed. **B**) Box-plot of α-diversity calculated with Chao1 and Shannon diversity indexes. Index comparison was performed using the Wilcoxon Rank Sum test. **C**) Two-dimensional principal coordinates analysis (PCoA) plot based on the Bray-Curtis dissimilarity matrix. Differences in β-diversity were estimated by permutational multivariate analysis of variance (PERMANOVA) after testing for sample dispersion. **D**) Differentially abundant amplicon sequence variants (ASVs) between healthy and epileptic dogs identified by DESeq2. ASVs with a log_2_ fold change > 2 or < 2 and adjusted p-values (q-values) > 0.05 were considered significantly associated with the health status, while only those with a log2 fold change > 3 or < 3 are shown in the volcano plot

Faecal microbiota analysis revealed the dominance of *Firmicutes* (37.1%), *Fusobacteria* (28.9%), and *Bacteroidetes* (22.5%) in all dogs. Differences in the mean relative abundance of bacterial genera were observed between IE and HC dogs (Fig. 2A). *Faecalibacterium*, *Prevotella*, *Megasphaera*, *Phascolarctobacterium*, and *Collinsella* were more abundant in HC dogs, while *Bacteroides*, *Megamonas*, *Escherichia-Shigella*, *Pseudomonas*,* Clostridium sensu strictu* 1, and *Enterococcus* spp. were more prevalent in IE dogs (Table 1). Furthermore, HC dogs showed a significantly higher bacterial richness (Chao1 index, *p* < 0.05) and a tendency towards higher bacterial evenness (Shannon index, *p* = 0.081) compared to IE dogs (Fig. 2B). Bray-Curtis dissimilarity was not significant (*p* = 0.18) (Fig. 2C), with the health status explained the 3.5% of the total variance of the faecal microbial community composition.

**Table 1:** Mean relative abundance (%) ± SEM of the most abundant genera identified in idiopathic epileptic (IE) and healthy dogs

Genus	Healthy dogs	IE dogs
*Fusobacterium*	27.9 ± 4.8	29.4 ± 4.4
*Faecalibacterium*	16.1 ± 3.3	10.2 ± 3
*Bacteroides*	5.8 ± 1.4	11.5 ± 3
*Prevotella*	10.4 ± 0.0	4.1 ± 0.0
*Collinsella*	7.6 ± 3.2	5.6 ± 1.8
*Alloprevotella*	5.7 ± 2.1	7 ± 2.4
*Megamonas*	2.8 ± 1.3	6.9 ± 2.5
*Blautia*	3 ± 0.8	3.3 ± 1.6
*Ruminococcus gnavus group*	2 ± 0.0	3.4 ± 0.0
*Peptoclostridium*	1.8 ± 0.5	1.7 ± 0.6
*Phascolarctobacterium*	2.1 ± 0.7	1.3 ± 0.5
*Sutterella*	1.5 ± 0.4	1.1 ± 0.4
*Clostridium sensu stricto 1*	0.1 ± 0	2 ± 1.2
*Megasphaera*	2.1 ± 1.1	0 ± 0
*Escherichia-Shigella*	0 ± 0	2 ± 1
*Lachnoclostridium*	1.2 ± 0.4	0.6 ± 0.1
*Pseudomonas*	0 ± 0	1.8 ± 1.2
*Anaerobiospirillum*	0.7 ± 0.2	1 ± 0.7
*Enterococcus*	0 ± 0	1.4 ± 1.0

DESeq2 analysis revealed numerous ASVs that were differentially abundant between IE and HC dogs, with the most significant taxa illustrated in Fig. 2D. Thirty and 26 ASVs were significantly abundant in HC and IE dogs, respectively (Supplementary Table S2). Specifically, ASVs assigned to *Prevotella* spp. (*n* = 7), *Blautia* spp. (*n* = 3), *Faecalibaterium* spp. (*n* = 1), *Phascolarctobacterium* spp. (*n* = 1), *Megasphaera elsdenii* (*n* = 1), *Ruminococcus torques group* spp. (*n* = 1), and *Succinivibrio* spp. (*n* = 1) were associated with HC dogs, while ASVs assigned to *Bacteroides* spp. (*n* = 5), *Alloprevotella* spp. (*n* = 4), *Megamonas* spp. (*n* = 4), *C. perfringens* (*n* = 3), *Escherichia-Shigella* group (*n* = 3), *Fusobacterium mortiferum* (*n* = 3), and *Clostridium sensu stricto* 1 spp. (*n* = 1) were associated with IE dogs (Supplementary Table S2).

## Discussion

We compared the faecal microbiota of antiepileptic drug-naïve IE dogs with that of a randomly selected HC group. Our 16 S rRNA analysis revealed loss of beneficial bacterial genera, overgrowth of opportunistic pathogens such as *Escherichia coli* and *C. perfringens*, and a reduction in bacterial diversity in the faecal microbiota of the IE group, consistent with the definition of dysbiosis proposed for companion animals [28]. Specifically, beneficial SCFAs-producing bacteria such as *Faecalibaterium* spp., *Blautia* spp., *Phascolarctobacterium* spp., *Ruminococcus* spp., *Megasphaera* spp., and *Prevotella* spp. [1, 29] were deficient in IE dogs and enriched in HC dogs. These findings align with human studies, which report reductions of *Prevotella*, *Faecalibaterium*, *Blautia* and *Ruminococcus* in epileptic patients compared to healthy controls [30, 31], supporting the role of SCFAs in the positive modulation of epileptogenesis [9, 32]. It has been hypothesized that GM-derived SCFAs exert neuroprotective effects via the gut-brain axis by straightening the tight junctions, promoting development, maturation and function of the astrocytes and the cerebral innate immune response cells, and reducing the local production of pro-inflammatory cytokines (i.e. IL-1-β, IL-6 and TNF-α), thereby lowering neuroinflammation [9, 33, 34]. This hypothesis suggests that gut inflammation and reduced circulating SCFAs driven by dysbiosis may promote neuroinflammation and increase seizures susceptibility [33–35].

The activation of Th-17 lymphocytes in the gut also plays a crucial role in epileptogenesis by releasing IL-17, which in turn elevates circulating IL-6, IL-1-β and TGF-α levels [9, 36]. These pro-inflammatory cytokines can infiltrate the brain, exacerbating BBB disruption, neuroinflammation and lowering the epileptogenic threshold [34, 35]. In our study, ASVs assigned to *Escherichia-Shigella* (*n* = 2) and *E. coli* (*n* = 1), known to induce Th-17-mediated immunity [37], were highly associated with IE dogs. In human drug-naïve epileptic patients, *Escherichia-Shigella spp*. abundance is higher than in healthy controls, and decreases after antiepileptic treatment [30]. Additional, other ASVs enriched in the IE dogs included *Bacteroides* spp., *Alloprevotella* spp., *Clostridium perfringens*, and *Fusobacterium mortiferum* (15 ASVs in total), all of which have been positively correlated with epilepsy in children [38]. Notably, *Fusobacterium mortiferum* is hypothesized to contribute to epileptogenesis through its metabolites, which may induce oxidative stress, neural apoptosis, and disruption of the BBB tight junctions [38–40]. *Bacteroides fragilis* and *Bacteroides dorei* are also implicated in Th-17 lymphocytes activation and BBB disruption, thereby contributing to neuroinflammation and epilepsy [38, 41].

To date, two studies investigate the differences in faecal microbiota of IE dogs and healthy controls [20, 21]. The study of García-Belenguer et al. [21] compared faecal microbiota composition of 10 IE mixed-breed dogs before and after antiepileptic treatment administration, with the microbiota from 12 healthy beagles fed with the same commercial dry food diet. The α-diversity (bacterial richness and evenness) did not differ between untreated IE dogs and healthy beagles, whereas their community composition (β-diversity) was significantly different. No differences in both α- and β-diversity between groups were observed by Muñana et al. [20], who compared the faecal microbiota of 13 pairs of epileptic and healthy dogs from the same household and on the same diet. In contrast to these two previous studies, we observed a remarkable trend towards a lower α-diversity in IE dogs, highlighting the importance of proper randomization of case and control dogs in microbiome studies. Differences in β-diversity results between the three studies might be influenced by the small sample size, likely reflecting variations in breed, diet and housing conditions rather than the epileptic status of the dogs.

As the correlation between GM and epilepsy has been widely considered, a scientific interest in potential therapeutic interventions in addition to standard anti-epileptic therapies, aiming at modifying the GM composition and at improving the clinical outcome in IE dogs, has grown recently. In addition to standard anti-epileptic drugs, dietary supplementation with medium-chain fat acids has shown a positive impact on seizure control and IE management [17, 18]. Although the mechanism has not been elucidate yet, it has been hypothesized that this diet supplementation may modulate GM composition and metabolism (i.e. SCFAs production) and thus resulting in improved energy metabolism and normal neuronal signalling in the brain [18, 19]. More recently, faecal microbiota transplantation has been proposed as intervention to modulate the GM composition of IE dogs [42]. Using anti-epileptic drug sensitive dogs without behavioural disorders as donors, this approach has been demonstrated to be effective in the management of fear and anxiety-like behaviours in epileptic dogs [42].

We acknowledge some limitations of our study. First, the small sample size, resulting from strict inclusion criteria, necessitates further trials with larger cohort of dogs to validate our findings. Second, although age, breed, BCS, dietary regimen, and reproductive status were not associated with differences between IE and HC dogs, indicating appropriate randomization, there was a predominance of male dogs in the IE group. While a male predisposition to idiopathic epilepsy in certain breeds has been reported [43], no sex-based differences in the faecal microbiota have been documented to date. Another limitation is the lack of standardization in the dietary regimen of both IE and HC dogs. As suggested by García-Belenguer et al. [21], using an IE and HC dogs fed the same diet could have reduced bias resulting from dietary variability. However, given that diet is generally recognised as one of the most important GM-shaping factors [44], enrolling animals fed with a heterogeneous commercial diet may better reflect the actual GM composition of the broader canine population, and support the generalizability of the alterations in bacteria diversity and abundance found in IE dogs observed in our study.

## Conclusions

Dogs diagnosed with IE exhibited a dysbiotic faecal microbiota characterized by reduced microbial diversity, a decrease in SCFAs-producing bacteria and an enrichment of opportunistic pathogens and Th-17-modulating bacteria. This finding correlates to the known neuroprotective and anti-inflammatory effects of microbial SCFAs and to the documented capability of Th-17 lymphocytes to trigger neuronal excitability, thus opening new scenarios on the etiopathogenesis of the canine idiopathic epilepsy. Furthermore, similar changes in intestinal microbiota of IE dogs and humans suggest that future studies on GM-modulating dietary interventions could position dogs as a valuable experimental model for investigating the treatment of epilepsy in humans.

## Electronic supplementary material

Below is the link to the electronic supplementary material.

Supplementary Material 1: Additional file 1: Table S1 and S2 reporting patient information and DeSEq2 results, respectively.

## Data Availability

No datasets were generated or analysed during the current study.
